# Myocardial segment-specific model generation for simulating the electrical action of the heart

**DOI:** 10.1186/1475-925X-6-21

**Published:** 2007-06-05

**Authors:** Darren A Hooks

**Affiliations:** 1 Bioengineering Institute, University of Auckland, New Zealand

## Abstract

**Background:**

Computer models of the electrical and mechanical actions of the heart, solved on geometrically realistic domains, are becoming an increasingly useful scientific tool. Construction of these models requires detailed measurement of the microstructural features which impact on the function of the heart. Currently a few generic cardiac models are in use for a wide range of simulation problems, and contributions to publicly accessible databases of cardiac structures, on which models can be solved, remain rare. This paper presents to-date the largest database of porcine left ventricular segment microstructural architecture, for use in both electrical and mechanical simulation.

**Methods:**

Cryosectioning techniques were used to reconstruct the myofibre and myosheet orientations in tissue blocks of size ~15 × 15 × 15 mm, taken from the mid-anterior left ventricular freewall, of seven hearts. Tissue sections were gathered on orthogonal planes, and the angles of intersection of myofibres and myosheets with these planes determined automatically with a gradient intensity based algorithm. These angles were then combined to provide a description of myofibre and myosheet variation throughout the tissue, in a form able to be input to biophysically based computational models of the heart.

**Results:**

Several microstructural features were common across all hearts. Myofibres rotated through 141 ± 18° (mean ± SD) from epicardium to endocardium, in near linear fashion. In the outer two-thirds of the wall sheet angles were predominantly negative, however, in the inner one-third an abrupt change in sheet angle, with reversal in sign, was seen in six of the seven hearts. Two distinct populations of sheets with orthogonal orientations often co-existed, usually with one population dominating. The utility of the tissue structures was demonstrated by simulating the passive and active electrical responses of two of the tissue blocks to current injection. Distinct patterns of electrical response were obtained in the two tissue blocks, illustrating the importance of testing model based predictions on a variety of tissue architectures.

**Conclusion:**

This study significantly expands the set of geometries on which models of cardiac function can be solved.

## Background

Computer modelling of the heart's electrical and mechanical action is a rapidly maturing field of study. Whole-heart and tissue-segment modelling of the time-dependent spread of electrical activation, and mechanical contraction [1], relies on realistic mathematical representations of both cardiac anatomy, and microstructure [2,3]. Acquiring geometric data for these models is a time-expensive process, and currently a small number of representative anatomical models [2-9] are in use internationally for a very wide range of simulation problems, encompassing the modelling of cardiac sinus rhythm, ventricular fibrillation, shock application, and drug-tissue interactions. Although many scientifically and clinically useful hypotheses can be tested in the modelling environment, additions to the set of realistic cardiac geometries, that are publicly available for use in simulations, are infrequent.

Ventricular myocardium has been shown to have a complex laminar structure, in which myocytes are grouped by perimysial collagen into branching sheets approximately four cells thick [7,10-15]. Extensive planes, across which myocyte-to-myocyte coupling is sparse, course through the ventricular wall, and have been termed "cleavage planes" [10]. Within this laminar organisation, myocyte axis rotates through some ~120° from epicardium to endocardium [10,12], and sheets are organised in a predominantly radial (transmural) orientation [10,15]. The most detailed anatomical models of the heart describe the spatial variations in both myo-fibre axis and myo-lamina (or "myo-sheet") orientation throughout the model domain [2,3,15,16]. At every location in these models, three angles, namely the fibre, sheet, and imbrication angles, determine the orientation of the local microstructural axes upon which tissue material properties can be defined [1]. Development of cardiac models carrying this detailed description of microstructure has been particularly time consuming.

This paper presents the development of a database [17] of tissue architecture built from small (~15 × 15 × 15 mm) myocardial blocks taken from the left ventricular (LV) freewall of seven porcine hearts. The laminar organisation of the tissue is reconstructed for each heart using a sequence of established techniques [10,15,18] involving cryosectioning of tissue on three orthogonal planes, with several modifications. The tissue models are compatible with use in simulations of either the mechanical or electrical action of segments of myocardium. Utility of the models is demonstrated with simulations of both the active (propagated) and passive electrical responses to current injection within the tissue volumes. This study contributes the largest database of porcine LV segment microstructure that is available for use by the wider cardiac research community.

## Methods

### Part I: tissue specific model generation

The model construction methods are based on those used by Costa et al. [15], and LeGrice et al. [10,11], with several modifications. All hearts used in model development were acquired following in-vivo electrophysiological measurement made in open chest pigs of weight 42–60 kg. The models were specifically developed to aid comparison of patterns of electrical propagation measured by an array of plunge electrodes in the in-vivo heart, and those predicted by a bidomain computational model of electrical activation [19]. All animal procedures were carried out under approval of the University of Auckland Animal Ethics Committee. In each animal, the heart was arrested by injection of cardioplegic solution into the LV, and then rapidly excised from the animal. The coronary arteries were flushed of blood using chilled cardioplegic solution, and electrodes replaced with styrene rod markers. The heart was then fixed by slow injection of 3% formalin in phosphate buffer solution simultaneously into all three coronary arteries.

**Figure 1:**
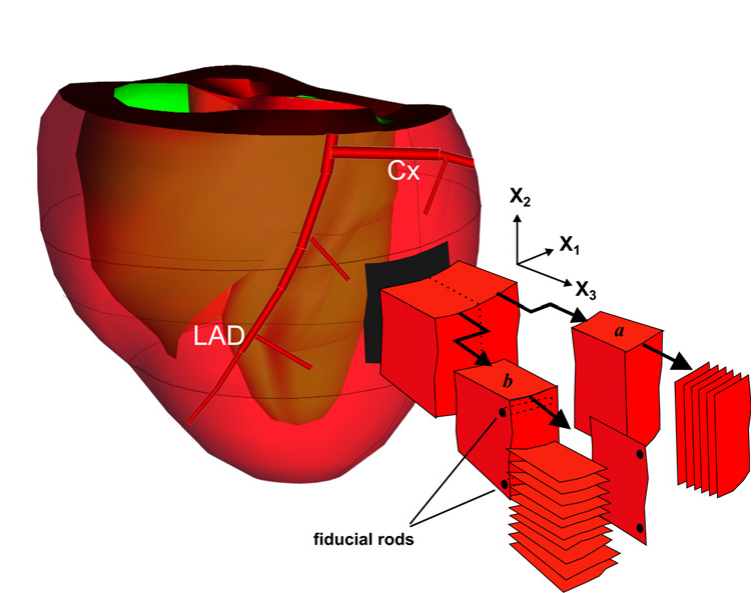
**Schematic of the tissue sectioning regime in relation to cardiac coordinates X_1_, X_2_, X_3_**. A block of anterior LV freewall myocardium is cut from the heart, and divided into two smaller blocks *a *and *b*. The block originates from the region of watershed between the left anterior descending (LAD) and circumflex (Cx) artery supply territories. Sections are taken from block a in the epicardial (X_1_-X_2_) plane, and from block b in both the base-apex (X_2_-X_3_) and circumferential (X_1_-X_3_) planes. Registration of sections from block b is aided by placement of fiducial rods prior to sectioning.

A cardiac coordinate system (X_1_, X_2_, X_3_) was defined as aligned with the local circumferential (X_1_), longitudinal (X_2_), and radial (X_3_) axes of the LV (Figure 1). The block of tissue to be modelled is excised from the mid-anterior LV freewall, by cutting in the X_2_-X_3 _and X_1_-X_3 _planes (Figure 1). The block centres were located from the apex between one-third and one-half the distance from the apex to the atrioventricular sulcus, and in the area of watershed between the left anterior descending and circumflex artery supply territories. Typical block sizes were ~15 × 15 × 15 mm. The block is then bisected longitudinally to yield two blocks (*a *and *b*, see Figure 1). These blocks are frozen rapidly in a cryostat (Leica; CM1510) cooled to -35°C. At this stage, distance measurements between adjacent styrene rods, marking the in-vivo electrode locations along the longitudinal (X_2_) axis, could be used to assess the degree of tissue shrinkage sustained during the fixation and freezing processes. Geometrical measurements made in the frozen tissue were later scaled in an isotropic fashion according to these measurements.

Using the cryostat, frozen sections of tissue (20 μm thickness) are then taken from both tissue blocks, transferred onto glass slides, and photographed. Serial sections in the plane of the epicardium (X_1_-X_2 _plane) are collected every 500 μm from block *a*, and used to characterise the variation in myofibre angle throughout the ventricular wall. From block *b*, a single slice is taken in the base-apex (X_2_-X_3_) plane, followed by serial sections (collected every 200 μm) in the circumferential (X_1_-X_3_) plane (Figure 1). The base-apex section, and the series of circumferential plane sections, all intersect myolaminae, the angles of which can be measured relative to the cardiac coordinate system. Registration of circumferential plane sections to the base-apex section was aided by the placement of two fiducial rods through block *b *in the X_1 _direction (Figure 1). It was found that much finer resolution of myolaminal organisation was possible with 20 μm thick sections compared to thicker (0.1–1 mm) sections used in previous studies [11,15].

**Figure 2:**
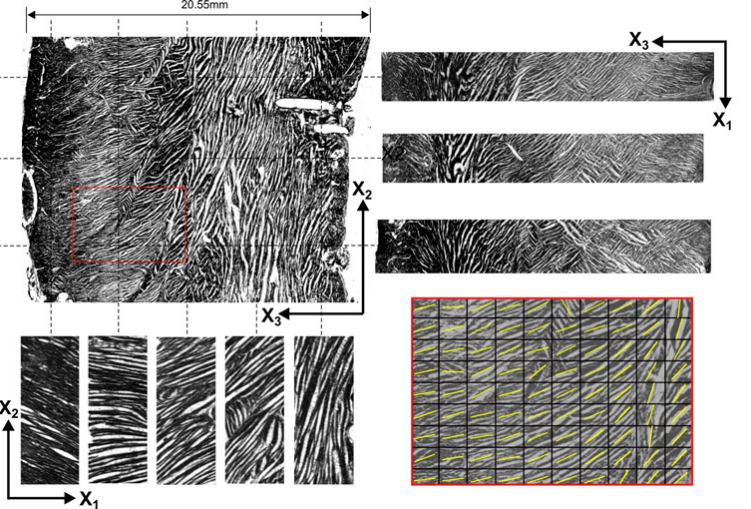
**Sample of tissue sections from tissue block EX07**. *Upper left*: Base-apex plane (X_2_-X_3_) section showing the orientation of myolaminae throughout the ventricular wall. *Upper right*: Circumferential plane (X_1_-X_3_) sections taken at three X_2 _locations through the wall. *Lower left*: Epicardial (X_1_-X_2_) plane sections taken at five X_3 _locations, revealing the gradual change in myofibre orientation from epicardium to endocardium. *Lower right*: Inset from the *upper-left *base-apex section showing zoomed-in section of tissue with overlayed structural angles automatically determined by the gradient-intensity algorithm.

Tissue sections from one processed myocardial tissue block (Ex07) are shown in Figure 2. The base-apex (X_2_-X_3_) plane slice is shown at upper-left. To the right of this section are three representative circumferential (X_1_-X_3_) plane slices, registered to the base-apex slice at three X_2 _locations. Beneath are five epicardial (X_1_-X_2_) plane sections showing fibre angle orientations at different transmural depths in the tissue. Microstructural angles can be measured from sections in each of the three planes. Under the assumption that myofibres run in-plane to the epicardium (imbrication angle is zero), angles measured in the epicardial plane slices represent myofibre orientation. Angles measured on the base-apex and circumferential plane slices represent the local angle of intersection of myolaminae with the slice plane. Microstructural angles are determined in each plane automatically from tissue section images using a gradient intensity algorithm [20,21]. Applied to the base-apex plane section, the algorithm was used to determine angles on a 30 × 30 grid (Figure 2; lower-right inset). Each circumferential plane section was similarly processed to obtain a set of 30 angles along the edge of the tissue that abutted the base-apex plane section. A single microstructural angle was computed for all epicardial plane sections. Following notation used previously [15], the microstructural angles measured on epicardial plane sections are termed α, those measured on the base-apex slice are termed β', and those on the circumferential plane slices β". Fibre angles (α) were measured relative to the cardiac X_1 _axis, whilst β' and β" angles were both measured relative to the X_3 _axis. Angles were signed positive or negative in accordance with previously developed convention [15].

The model allowed variation in α along the X_3 _direction, whilst β' and β" both varied in X_2 _and X_3 _directions.

Myolamina orientation was usually difficult to discern from base-apex and circumferential sections immediately adjacent to the epi- and endo-cardium, where coupling of adjacent laminae is known to be tightest [14,22]. In these areas, and where blood vessels obscured the laminar structure, the microstructural angle β' or β", computed by the gradient intensity algorithm, was manually rejected.

**Figure 3:**
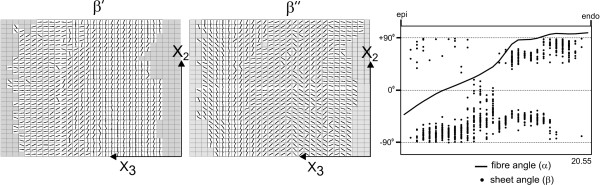
**Model description of tissue block EX07**. *Left panel*: Cleavage plane angles (β') computed for the base-apex section shown in Fig. 2 *upper-left*, on a 30 × 30 grid. *Middle panel*: Cleavage plane angles (β") computed from serial circumferential plane (X_1_-X_3_) sections, mapped to the same base-apex plane as in *A*. Each row of angles is derived from a single circumferential plane tissue section, along its edge that abuts the base-apex plane section. Grayed boxes in the grids of both β' and β" panels represent areas of indeterminate cleavage plane angle. *Right panel*: Graph of the full model description including the transmural dependence of myofibre angle (line), from epicardium (epi) to endocardium (endo), and transmural distribution of sheet angles (β; dots) as derived from the β' and β" fields. The transmural thickness of the tissue block (20.55 mm) is shown at the bottom-right corner of the graph.

Complete sets of β' and β" angles are shown for the same tissue block (Ex07) in Figure 3. Areas where microstructural angles could not be determined confidently are shown in grey. The set (30 × 30 elements) of β' angles (Figure 3; left panel) are derived directly from the base-apex plane image shown in Figure 2 (upper-left panel). The corresponding set of β" angles (Figure 3; middle panel) consists of 30 rows each of which is derived from a single circumferential plane tissue section, along its edge that abuts the base-apex plane section.

A continuous description of fibre angle variation through the wall was generated by fitting 10 linear finite elements to the fibre angle data (Figure 3; right panel). Fitted fibre angles at a given transmural depth (X_3 _location) could then be combined with measured β' or β" angles at the same transmural depth to derive sheet angles (β) at that depth, using the formulae [15]:

tan β = tanβ'cosα   (|α| ≤ 45°)

tan β = -tanβ"sinα   (|α| > 45°)

Equation 1 was applied where the absolute fibre angle was ≤ 45°, as in this range β' provided the most accurate projection of sheet angle, whilst equation 2 was applied outside this range, where β" provided the most accurate projection [15]. The final tissue model (Figure 3; right panel) consisted of the continuous fibre angle description across the wall, and up to 900 discrete sheet angles (β) mapped to locations in the base-apex plane. The discrete description of sheet angles allowed accurate representation of discontinuities in sheet angle across the wall.

### Part II: application to electrical simulation

The electrical action of the heart can be best represented by a bidomain model in which the intra- and extra- cellular spaces of the heart are conceptualised as interpenetrating, and extending throughout the entire model volume. The governing equations of the model [3,19] are solved within the CMISS [23] computational framework using a number of different techniques, however, for this study, a grid-based finite element solution technique is used as previously described [3,24]. This solution technique allows the piecewise discontinuous incorporation of myofibre and myosheet angles on an element-by-element basis over the solution domain.

Current of 0.03 mA is withdrawn from the extracellular space of two example model tissues, at a point located centrally in the model volume (~8.5 mm below the epicardium). Equal magnitude (but opposite sign) current is uniformly distributed amongst all the tissue boundaries except for the epicardium, to match the experimental case of recordings taken from an open chest pig. The steady-state extracellular potential (*Φ*_*e*_) field generated by the current sink (cathode) is simulated first in the absence of any cellular response to the current. Subsequently, the actively propagated wavefront generated by the cathodal current is examined by simulation of the activation time (*AT*) field over the model volumes. A simple cubic model of the cardiac action potential is utilised for the active models [3,25]. The conductivities used in the models (chosen in approximate accordance with previous work) [3] are (in units of S/m) *g*_*il *_= 0.263, *g*_*it *_= 0.0263, *g*_*in *_= 0.008, *g*_*el *_= 0.263, *g*_*et *_= 0.245, and *g*_*en *_= 0.1087. Membrane capacitance of the models was set to 0.01 μF/mm^2^, and membrane conductivity to 0.004 mS/mm^2^. The cubic action potential model had a resting potential of -85 mV, a threshold potential of -80 mV, and a plateau potential of 15 mV.

## Results

### Part I: tissue specific model generation

**Figure 4:**
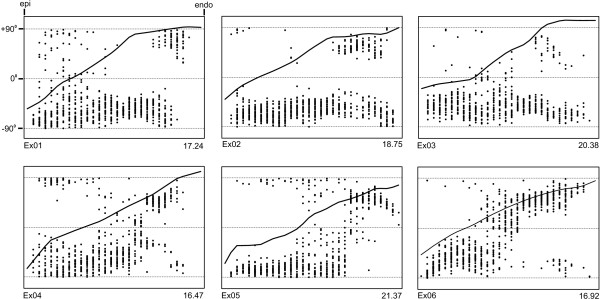
**Model geometries from heart blocks Ex01-Ex06**. Each graph shows the transition in fibre angle through the heart wall, from epicardium (left) to endocardium (right), plotted between -90° to +90° (upper and lower horizontal dashed lines respectively). The transmural depth of each tissue block is recorded in *mm *at the bottom right of each graph.

The model geometries reconstructed from the remaining six (Ex01-06) heart segments, each from a different pig, are presented in Figure 4. Each graph shows the variation of fibre and sheet angles across the wall from epicardium to endocardium, and gives the total transmural wall thickness (in mm) of each sample at the bottom-right of the graph. From these graphs, and that shown in Figure 3, several observations, general to all the hearts examined, can be made. Firstly, it is clear that across all hearts, myofibres rotate in near linear fashion from epicardium to endocardium. In general, the rate of change of fibre angle with respect to transmural depth is least towards the endocardial surface, with the exception of Ex03 where a section of slow change in fibre angle is observed through the first 25% of the wall from epicardium. Fibre angles averaged -49.7 ± 17.9° (mean ± SD) at the epicardium, and 91.6 ± 8.4° at the endocardium. In all hearts, the cross-over point of fibre angle from negative to positive was located closer to the epicardium than the endocardium. The cross-over depth as a percentage of wall-thickness averaged 30.6 ± 9.2 (mean ± SD). In the outer two-thirds of the wall sheet angles were predominantly negative, with the presence of some interspersed positive angles. In the inner one-third of the wall an abrupt change in sheet angle was seen in six of the seven hearts. In five of these six hearts, the predominant sheet orientation reversed in sign towards the endocardium. In one heart (Ex02), two distinct populations of positively and negatively angled sheets co-existed in approximately equal proportion, towards the endocardium.

Measurements of electrode locations preceding and following the fixation and freezing processes revealed that the tissue blocks shrunk on average 10 ± 8% (mean ± SD) along the longitudinal (X_2_) axis.

All seven tissue segment models are available on the web [17] for research purposes.

### Part II: application to electrical simulation

**Figure 5:**
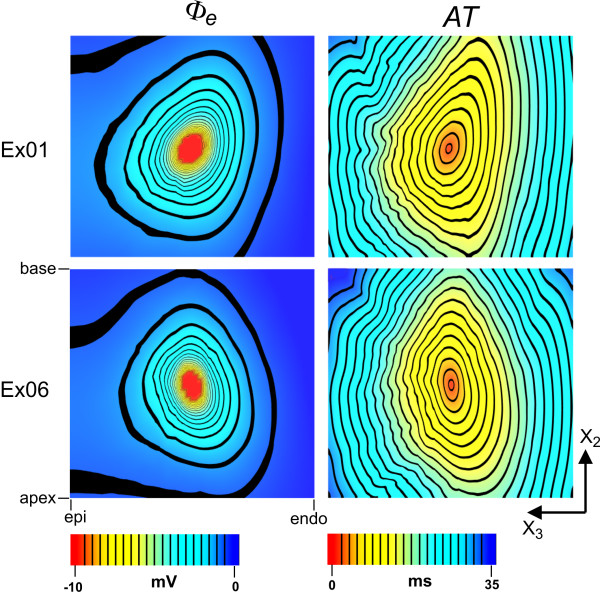
**Example simulations**. Examples of simulations using model volumes Ex01 (*upper panels*) and Ex06 (*lower panels*). Extracellular potential fields (*Φ*_*e*_) generated by focal current application at the tissue centres are shown in the *left panels*. Activation time (*AT*) fields derived from wavefront propagation from the site of current injection are shown in the *right panels*. The epi (epicardium) to endo (endocardium) distance is ~17 mm for both models.

The results of the passive and active bidomain simulations are shown in Figure 5, for two example tissue blocks (Ex01 and Ex06). Extracellular potential (*Φ*_*e*_) and activation time (*AT*) fields are shown on the central base-apex plane for both tissues. Figure 5 demonstrates the dependence of cardiac behaviour on structure, and therefore the importance of testing computer model based predictions on a variety of tissue architectures. The two tissue blocks give rise to quite distinct patterns of *Φ*_*e *_and *AT*, with anisotropy in the fields being aligned with the microstructural axes of each tissue model. Predominantly negative sheet angles in the vicinity of the stimulus site in Ex01 determine the bottom-left to top-right slant of the fields for this tissue. Conversely, Ex06 exhibits predominantly positive sheet angles in the same region, and the fields for this tissue are accordingly slanted in the opposite direction.

## Discussion

The last two decades have seen a dramatic increase in the use of computational models of the electrical and mechanical action of the heart on geometrically realistic model domains. One of the first studies to incorporate realistic anatomical features into a model environment solved electrical propagation on a three-dimensional network of cardiac fibres reconstructed from histological sections [26]. Later, an anatomically realistic finite element structural model of the canine ventricles was developed which incorporated both fibre and sheet fields at ~14,000 points throughout the ventricular walls [2]. This model was constructed using a range of standard histological techniques, and required weeks to collect the necessary data [10]. The resulting finite element model was tailored to the solution of both mechanical and electrical problems. More recently, such work has been extended by a number of groups. One of the most intensively used anatomical models currently is that of the rabbit heart [5], which by virtue of its smaller scale compared to the canine heart model, allows for the solution of mechanical and electrical problems in a more realistic time-frame [27], but with the cost, at times, of diminished realism in relation to the human heart. Increasingly, MRI has been used as a tool to more rapidly generate mathematical descriptions of cardiac anatomy [4,8,9,28]. When combined with diffusion tensor MRI (DTMRI) these scans have the potential to reconstruct both fibre [7] and sheet [7,29] fields at reasonable resolution, with much less effort than traditional histological methods. Scan times of some 60 hours afford high resolution of structural details at voxel sizes of ~300 μm in plane, and ~800 μm out of plane [4]. Recent models constructed by use of MRI include models of human atria [8,9] which have been used to aid optimisation of the clinical procedure of percutaneous atrial fibrillation ablation [9]. Techniques to deform ventricular models from MR images to template geometries also promise to yield comparisons in sheet and fibre fields in a variety of pathological states [4]. Recently "extended volume" confocal microscopy, another truly three-dimensional imaging modality [30], has been used in the development of models of small segments of rat LV myocardium [3,14]. These models have contributed understanding of the mechanisms involved in successful ventricular defibrillation [3,16].

**Figure 6:**
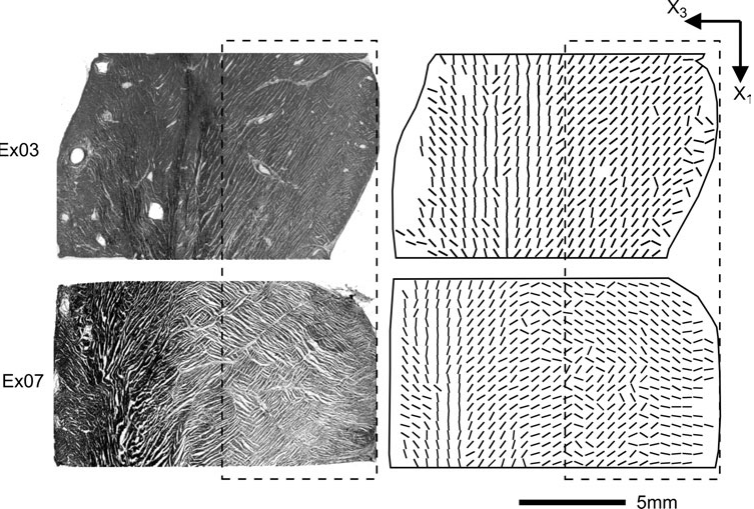
**Structural variation in the circumferential direction**. Circumferential (X_1_-X_3_) plane sections shown from two hearts to demonstrate the dependence of sheet angle on X_1 _location. Dashed box encompasses the approximate region where fibre angle is >45° – where sheet orientations can be determined from X_1_-X_3 _plane sections with good accuracy. The right panels contain the automatically determined structural angles. Tissue block Ex03 demonstrates constant sheet orientations over 10 mm in the X_1 _direction, whilst the predominant sheet angle in Ex07 reverses through 90° over the same distance.

In this study traditional cryo-sectioning techniques were chosen to allow reconstruction of a plane of tissue structure from blocks of porcine LV. The methods used require destructive tissue sectioning in three planes in order to build the tissue description of a single base-apex plane. In our case, the attraction of MRI in enabling full three-dimensional reconstruction of fibre and sheet angle fields was out-weighed by several factors. Although the case for the ability of DTMRI to accurately reconstruct cardiac fibre fields is compelling [6,7,29], its ability to reproduce sheet orientations with sufficient accuracy and resolution for our purposes, is less clear. The sole study to compare DTMRI derived sheet vectors with histological measurements in the same tissue [29] reports relatively wide distributions of error between the two methods (although most of this error is attributed to errors involved in the ink-staining method used in histological determination of angles). The study could not address the degree of correlation at scales finer than the ~3 mm resolution in the DTMRI scans. That is not to dispute that DTMRI measures of sheet angle remain highly correlated with measured angles in the same [29] and other hearts [7], and are likely sufficient for analysis of the function of the laminar organisation at the level of the whole heart [31]. In the first instance, we aim to use the tissue models presented here to compare with electrical data gathered from a plane of high-density plunge electrodes. High-resolution, accurate determination of the tissue structure adjacent to the electrode plane was therefore paramount in our selection of reconstruction technique. Whilst automated "extended-volume" confocal microscopy is capable of extremely high-resolution three-dimensional reconstruction of tissue architecture [30], it was not a viable approach for tissue volumes of the dimension considered here due to the lengthy imaging times involved (typically 1 week for a 1 mm^3 ^volume). The trade-off for using cryosectioning techniques is that to allow for modelling over a three-dimensional block of tissue, an assumption that the tissue structure is constant over small distances in the third (circumferential; X_1_) dimension must be applied. Support for such an assumption comes from the observation that myofibre angles generally vary little in the circumferential direction, over distances of 1–2 cm. However, the myosheet angle field tends to be more discontinuous than that of myofibres. The circumferential plane sections taken in this study give some indication of the variation in sheet angle field in the circumferential direction, in the region of the sections where absolute fibre angle is greater than 45°. In a qualitative sense, five of the seven hearts examined for this study displayed a reasonably constant sheet angle field in the X_1 _direction over 10 mm from the central base-apex plane. The other two hearts did contain significant variations in the field, and in one of these there was complete reversal of sheet angles within the |α|>45° range, over the distance of 10 mm. Figure 6 presents circumferential plane sections from (i) a representative block (Ex03) that showed little change in sheet orientation over 10 mm, and (ii) the block (Ex07) that showed the most change. In consideration of Ex07, the worst case, there will be some loss of realism when using the model geometry reported here, to solve models over a three-dimensional tissue domain.

The validity of equations 1 and 2 used in the model construction relies on the assumption that myofibres run in plane with the epicardium (X_1_-X_2 _plane), and hence imbrication angles (the angle subtended by the myocyte axis with the epicardial plane) are uniformly assumed to be zero. This assumption is supported by observations made in our lab of negligible fibre imbrication in the rat LV freewall [14], and historical measurements taken from human LV myocardium in which mean imbrication angles varied from -3.48° to -4.64° at two sites in the LV freewall [12].

Previous models of sheet architecture have used polynomial descriptions of both fibre and sheet angle variations through the model volume, and weighted contributions of β' and β" angles in the determination of sheet angle (β) [10,15]. The models presented here elect to use a piecewise discontinuous description of sheet angle, as this allows capture of the abrupt changes in sheet angle that occur throughout the wall. Discontinuous contributions of β' and β" to sheet angle are also applied (equations 1 and 2), to ensure that any given sheet angle is representative of the structural angle measured at a given location in one or other of the X_2_-X_3 _or X_1_-X_3 _plane sections. Much of the scatter present in our graphs of sheet angle (Fig. 4) is due to the regular co-existence of two distinct population of sheets oriented orthogonally to each other. Usually this takes the form of a dominant population which is interspersed with smaller pockets of orthogonal sheets (see the circumferential sections of Fig. 2 for excellent examples), however in cases such as towards the endocardium of Ex02, the two populations can approach equal weighting. This observation parallels the sheet angle measurements of Arts et al. [18], who also found the existence of two sets of sheet populations, usually with a dominant population. These population sets were shown to be oriented along planes of maximum systolic shear strain, and therefore contribute to wall-thickening during systole [11].

Comparison between our tissue structures, and those determined in dog hearts at very similar location [15], can be made. The data of Costa et al. [15] (see their Figs. 4 and 5) mirrors our findings that (i) fibre angle varies approximately linearly from epi- to endocardium, and (ii) sheet angles are predominantly negative in the midwall of the mid-anterior LV freewall, but have greater variance of angle closer to the endocardium, in some hearts reversing in orientation in this region. Recent observation has been made in swine right ventricular tissue of an abrupt change in myofibre angle through some ~60° (average rate of change ~1700°/mm), at an average depth of ~500 μm from the epicardial surface [21]. Whilst in two of the seven hearts (Ex04 and Ex05; Fig. 4) in this study fibre angle did rotate most rapidly in the subepicardium, in general such an abrupt change in angle was not observed, despite collecting sections at 50 μm intervals within the first few millimeters of wall thickness. This discrepancy likely reflects a difference in the architecture of the right and left ventricles.

Transmural fibre variation was also measured by Streeter and Bassett, in the left ventricles of six pig hearts [32]. Whilst a near linear rotation of fibre angle was observed across the wall in the lateral LV freewall, measurements from the anterior LV freewall showed significant departures from linearity, and a much larger degree of scatter in angles [32]. Our fibre angle data is most consistent with that shown for the lateral LV freewall. In that region, the difference in fibre angles measured on the epicardium and endocardium ranged between 116° and 157° [32]. In comparison, the range of fibre rotation for our samples was 117° to 176° Interestingly, Streeter and Bassett comment on the presence of "occasional specialised fibre directions that abruptly diverge from the fibre flow of the continuum." Such divergent pockets of fibres were noted to contribute to the scatter of fibre angle shown toward the endocardium of the anterior LV freewall [32].

Occasional pockets of fibres with variant angle were observed in our tissue sections also, an example of which is seen in an epicardial (X_1_-X_2_) plane section of Figure 2 (see fourth section from the left), however they were not a major feature. Because the intensity gradient method employed for angle detection in this study estimated the dominant angle of any epicardial plane section, these divergent pockets had little or no impact on the fibre angle data presented here.

In summary, this study contributes the largest database of porcine LV segment microstructure that is available for use by the cardiac research community. The key limitations of this study are (1) the inability to measure changes in the laminar architecture in the circumferential axis of the tissue blocks, and (2) the reliance on the assumption of zero fibre imbrication angle. To address these limitations whilst preserving high spatial resolution of the laminar architecture, development of a new technique involving repetitive milling, etching, and staining, in a semi-automated fashion, through blocks of wax-embedded tissue, is underway in our laboratory, and promises to yield fully three-dimensional reconstructions of moderate sized tissue blocks in the future.
